# Characterizing changes in transcriptome and kinome responses in testicular cells during infection by Ebola virus

**DOI:** 10.1038/s44298-024-00022-8

**Published:** 2024-04-11

**Authors:** Andrew L. Webb, Brayden G. Schindell, Geoff Soule, Abu B. Siddik, Bernard Abrenica, Harram Memon, Ruey-Chyi Su, David Safronetz, Jason Kindrachuk

**Affiliations:** 1 Department of Medical Microbiology & Infectious Diseases, University of Manitoba, Winnipeg, MB Canada; 2 Special Pathogens Program, National Microbiology Laboratory, Public Health Agency of Canada, Winnipeg, MB Canada; 3 JC Wilt Infectious Diseases Research Center, National Microbiology Laboratory, Public Health Agency of Canada, Winnipeg, MB Canada; 4 Manitoba Centre for Proteomics and Systems Biology, Department of Internal Medicine, University of Manitoba, Winnipeg, MB Canada

**Keywords:** Virology, Immunopathogenesis, Biochemistry, Computational biology and bioinformatics

## Abstract

Ebola virus (EBOV) is able to persist and actively replicate in the reproductive tract of male disease survivors months or years after recovery from Ebola virus disease (EVD)^1^. Persistent EBOV infections are usually asymptomatic and can be transmitted sexually, but the host and viral factors that mediate these infections have not been characterized^2,3^. We investigated the interaction between host and viral factors during EBOV infection of the blood testis barrier (BTB), with a focus on Sertoli cells as a potential reservoir for viral persistence. We assessed viral replication kinetics and host responses of mouse testicular Leydig cells and Sertoli cells infected with EBOV Makona (i.e. infectious EBOV) and collected samples up to 28 days post-infection. Viral replication was apparent in both cell lines, but intracellular early viral loads were much higher in Leydig cells compared to Sertoli cells. We used RNAseq analysis to characterize transcriptomic responses of Leydig cells and Sertoli cells to EBOV infection over time. Further investigation of early interactions between host cells and EBOV was performed using virus-like particles (EBOV trVLP) and assays of phosphorylation-based cell signaling. Our findings indicate that virus-treated Sertoli cells responded more rapidly and robustly than Leydig cells, and with a particular emphasis on detection of, and response to, external stimuli. We discuss how the roles played by Sertoli cells in immune privilege and spermatogenesis may affect their initial and continued response to EBOV infection in a manner that could facilitate asymptomatic persistence.

## Introduction

Recently, Ebola virus disease (EVD) outbreaks have increased in frequency and duration^3^. The West African EVD epidemic of 2014–2016 resulted in more than 28,000 cases and 11,000 deaths, and outbreaks continue to occur despite increased awareness and implementation of containment and contact tracing protocols^4^. The frequency with which Ebola virus (EBOV) “spills over” directly from wildlife to human populations is unclear, but infected individuals are at high risk of beginning chains of transmission that can rapidly gain momentum^5,6^. While current treatment protocols dictate that EVD patients be discharged based on symptomology and blood tests, a high proportion of male survivors may harbor EBOV genetic material in their semen and reproductive tract long after convalescence^4,7^. These findings are noteworthy because EBOV can remain viable during persistent and asymptomatic infection of the reproductive tract, and cases of sexual transmission from male EVD survivors leading to new chains of transmission have been reported^8–11^. In this context, it is vital to characterize the factors that contribute to EBOV persistence in the male reproductive tract. While there is an abundance of data for persistence of EBOV genomic material in semen of male EVD survivors following recovery, there is little data regarding EBOV cell tropism within the testis, and the molecular factors that mediate persistent infections remain unidentified^3^.

Despite frequent detection of EBOV genomic material in semen, human survivors of EVD rarely report testis pain as a symptom of post-EVD syndrome^12,13^. However, there is little data regarding dissemination of EBOV into, and persistence within, the male reproductive tract. In particular, the lack of human tissue samples hinders investigation into EBOV replication and host response. Previous studies using animal models reported EBOV dissemination within the male reproductive tract^14–16^. Acute studies of EBOV infection in monkeys variously detected viral material in the interstitial and epithelial cells of the seminiferous tubules and the epididymis^14–16^. These studies note minimal immune response and organ pathology, which was primarily attributed to hemostatic dysfunction^14–16^. A study of EBOV persistence in monkeys found EBOV in the tubular lumen of the epididymis 43 days post infection (dpi) in one of eight survivors^17^. In this case, the presence of necrotic cellular debris and inflammatory cells was also observed in the interstitial space and tubular lumen^17^. In a mouse model of EBOV persistence in the male reproductive tract, viral RNA was detected in epididymal epithelial cells at 14 dpi and 35 dpi^18^. Evidence of spermatogonia and epididymal cell degeneration, as well as interstitial inflammation, was also noted to increase from 14 dpi to 35 dpi^18^. Although these animal studies corroborate EBOV persistence in the human male reproductive tract, little is known about the molecular mechanisms of interaction between EBOV and specific testicular tissues during infection.

Tissue cultures provide a means of observing the response of specific cell types to infection at lower costs and with fewer ethical considerations than animal models. While tissue culture experiments do not recreate many aspects of animal models, such as an interdependent host immune system, isolated cells can be used to determine viral tropism and replication kinetics, and to identify patterns of host cell response to infection. Whenever possible, use of infectious virus during EBOV experiments is ideal, but this necessitates containment level 4 protocols that can limit method feasibility. Transcription and replication competent virus like particles (trVLP) provide an alternative to infectious viruses and can be handled under lower containment level protocols^19^. In the current study, we used infectious EBOV and EBOV trVLP and two cell types associated with the blood testis barrier (BTB). Leydig cells are located in the interstitial space of the BTB, where their primary function is the regulation of spermatogenesis via growth factor and steroid signaling^20^. In particular, the binding of luteinizing hormone with luteinizing hormone receptors on Leydig cells stimulates the production of androgens such as testosterone, which in turn binds to receptors on Sertoli cells to propagate spermatogenesis^20^. Epithelial Sertoli cells form the structure of the BTB; they limit host inflammatory immune response, oppose pathogenic dissemination into the seminiferous tubules, and provide nutrients and signaling factors to developing germ cells^21,22^. Sertoli cells also phagocytose apoptotic germ cells to clear cellular debris and provide nutrients for further spermatogenesis^23^.

The objective of this study was to investigate the interaction between EBOV and specific types of cells associated with the BTB. We used infectious EBOV to determine whether the virus can infect and replicate within mouse Leydig cells and Sertoli cells. We also characterized changes in Leydig cell and Sertoli cell gene expression as EBOV infection progressed. Finally, we used EBOV trVLP treatments to identify changes in host cell signaling and membrane function in response to EBOV binding and entry. Our aim was to uncover putative host factors in EBOV infection and persistence as potential topics of future exploration.

## Methods

### Viruses and cell lines

EBOV *H.sapiens*-tc/GIN/2014/Gueckedou-C07 expressing green fluorescent protein was used for all infectious EBOV experiments, which was performed at the Canadian Science Center for Human and Animal Health, National Microbiology Laboratory. The wild-type genome is available on Genbank (Accession No. KJ660347.2), and we confirmed that the EGFP expression cassette was inserted between NP and VP35 prior to its use^24^. Mouse MLTC-1 (i.e. Leydig) cells were maintained in RPMI (Gibco; Billings, MT, USA) with 5% fetal bovine serum (FBS; Gibco) and 1X penicillin/streptomycin (Gibco). Mouse 15-P1 (i.e. Sertoli) cells were maintained in DMEM (Gibco) with 10% FBS (Gibco) and 1X penicillin/streptomycin (Gibco). Both cell lines were incubated in 5% CO_2_ atmosphere at 37 °C. All work with trVLPs were performed at the University of Manitoba in accordance with institutional biosafety policies. Work described with infectious EBOV was performed at the Canadian Science Centre for Human and Animal Health, Public Health Agency of Canada, in accordance with institutional biosafety policies.

### EBOV trVLP production

In order to investigate host cell responses to EBOV binding and entry under CL2 conditions, we used an established plasmid-based system to create EBOV trVLP to model infectious EBOV^19,25^. HEK 293T cells were seeded onto 6-well plates at 5 × 10^5^ cells per well in DMEM with 10% FBS and 1X penicillin /streptomycin. After 24 h, confluent cells were transfected with plasmids encoding genes for EBOV nucleoprotein (NP; 250 ng per well), viral protein 35 (VP35; 250 ng per well), L polymerase (L; 2000 ng per well), viral protein 30 (VP30; 150 ng per well), T7 polymerase (T7; 500 ng per well), and the tetracistronic minigenome (500 ng per well). The tetracistronic minigenome contains the firefly luciferase gene, viral protein 40, viral glycoprotein, and viral protein 24^19^. Plasmids were kindly provided by Dr. Thomas Hoenen (Friedrich-Loeffler-Institut; Greifswald, Mecklenburg-Vorpommern, Germany). Transfections were performed using 6 µl per well of FuGENE Transfection Reagent (Promega; Madison, WI, USA) according to the manufacturer’s specifications. Transfected cells were incubated at 37 °C and 5% CO2 in DMEM containing 10% FBS and 1X penicillin/streptomycin. After 24 h, cell media was replaced with fresh media containing 5% FBS and 1X penicillin/streptomycin at a volume of 2 ml per well. After a further 72 h, samples were collected from 3 wells of HEK293T control cells and EBOV trVLP-producing cells. Cell pellets were separated by centrifuge at 800 × *g* for 5 min and washed with PBS, then resuspended in Bright-Glo lysis buffer (Promega) and stored at −80 °C. Supernatants from each of HEK293T control cells and EBOV trVLP-producing cells were combined, and cellular debris was separated by centrifuge; three aliquots of the VLP-containing media were collected, from which RNA was extracted using the RNeasy Mini Kit (Qiagen).

### Infectious EBOV treatment and sample collection

Leydig cells or Sertoli cells were seeded onto 12-well plates at 1 × 10^5^ cells per well in DMEM with 10% FBS and 1X penicillin-streptomycin, and incubated in 5% CO_2_ atmosphere at 37 °C. After 24 h, cell culture media was replaced with fresh media containing 1% FBS for Leydig cells or 2% FBS for Sertoli cells and allowed to rest for a further 24 h. Cells were treated with fresh media alone, or with fresh media containing EBOV at 0.1 multiplicity of infection. After 1 h, cells were washed with PBS and media was replaced. During the experiment, media was replaced every 2 days. Three samples of supernatant and cell pellets were collected from separate wells for each time point, cell line, and treatment. Sample time points were 1, 2, 4, 7, 14, and 28 dpi, with mock-infected samples collected at each time point as controls. Samples were stored at −80 °C until the end of the experiment. RNA was extracted from tissue cell pellets and cell supernatants using the RNAeasy Mini kit as per the manufacturers’ instructions (Qiagen; Toronto, ON).

### EBOV trVLP treatment and sample collection

Leydig cells and Sertoli cells were seeded onto 12-well plates at 1 × 10^5^ cells per well in DMEM with 10% FBS and 1X penicillin-streptomycin, and incubated in 5% CO_2_ atmosphere at 37 °C. After 24 h, cell culture media was replaced with 1 ml per well of media from HEK293T control cells or EBOV trVLP-producing cells. After 24 h, media was replaced with 1 ml per well of fresh DMEM with 5% FBS and 1X penicillin-streptomycin. From separate wells, six cell pellets were collected at 1 h, 6 h, and 24 h post treatment for each cell line and treatment. Cell pellets were separated by centrifuge and washed with PBS. Three cell pellets were resuspended in TRIzol (Invitrogen; Burlington, ON, Canada) and three in Bright-Glo lysis buffer. Samples were stored at −80 °C until needed. After 24 h, remaining cell tissue cultures were washed with PBS and fresh media was added. Cells were incubated a further 48 h (i.e. 72 h post treatment) and a final set of samples were collected.

### Strand-specific reverse transcription PCR

We used strand-specific reverse transcription and real-time PCR to quantitate EBOV RNA based on detection of the VP40 gene. We reverse-transcribed 2 µg of RNA from each cell pellet sample using the High-Capacity cDNA Reverse Transcription Kit (Applied Biosystems; Waltham, MA, USA), for which we replaced the random primers provided in the RT-PCR kit with a primer specific to viral genome RNA (Table 1). The quantity of strand-specific primer was 10 pmol per RT-PCR reaction. Product from reverse-transcription PCR reaction was diluted five-fold, followed by qPCR with a forward primer specific to the tagged sequence and reverse primer specific to the EBOV VP40 gene (Table 1). Real-time PCR was performed using a QuantStudio 6 Flex Real-Time PCR System (Applied Biosystems) with PowerUp SYBR Green PCR master mix (Applied Biosystems).

**Table 1:** Sequences for primers used during reverse transcription and quantitation of RNA collected from infectious EBOV-treated and EBOV trVLP-treated Leydig cells and Sertoli cells

Primer target	Primer sequence (5’ to 3’)	PCR type	Tm (°C)
Strand-specific vRNA	GGCCGTCATGGTGGCGAATGGTGAATGTCATATCGGGCCC	Reverse transcription	62.5
Primer tag	GGCCGTCATGGTGGCGAAT	Real-time qPCR	65.1
VP40 (reverse)	GATGGCGGCCGTAGTTGAG		62.4

### Transcriptome analysis by RNAseq

We performed RNAseq for EBOV-infected and control Leydig cells and Sertoli cells. Sample library prep was performed using the True Seq Standard Total RNA Library Prep Gold kit (Illumina; San Diego, CA, USA) and sequence runs were performed using the NextSeq 500/500 High Output Flow Cell V2.5 cartridge (Illumina). The quality of sequence reads was increased using the R Trimmomatic tool and specifications for a sliding window average across four bases and a minimum quality of 20^26^. Trimmed reads were aligned to the reference genome mm10 using the R HISAT2 alignment tool with specifications for paired reads^27^. Gene annotation and feature count were performed using the mm10 reference genome and the R FeatureCounts tool restricted to paired fragments with both reads aligned^28^. Normalization and differential expression analysis were performed in tandem using the R DESEQ2 tool^29^. During this process, triplicate infected and mock-infected control sample dataset were converted to an individual dataset for each time point that represented differential gene expression of infected samples relative to their respective matched mock-infected time point. The online tool Metascape (https://metascape.org; accessed October 5, 2023) was used to perform pathway overrepresentation and gene ontology analysis for genes with log_2_ fold change greater than 2 or less than -2 (adjusted *P* ≤ 0.05)^30^. Results for gene list queries were limited to GO Biological Processes (https://geneontology.org; accessed October 5, 2023) and Reactome Gene Sets (https://reactome.org; accessed October 5, 2023)^31–34^.

### Protein phosphorylation assay

We characterized early host cell signaling activity in response to EBOV infection by analyzing kinase activity using a modified form of a previously described peptide array for kinome analysis^35^. Leydig cells and Sertoli cells were seeded onto 6-well plates at 5 × 10^5^ cells per well in DMEM with 10% FBS and 1X penicillin/streptomycin. After 24 h, the media of target cells was removed, and the media collected from HEK293T control cells or EBOV trVLP-producing cells was added at a volume of 3 ml per well. At 1 h post treatment and 6 h post treatment, cells collected from three wells were combined into one sample for each cell line and treatment. Cell pellets were separated from media by centrifuge at 800 × *g* for 10 min and lysed by resuspension in 100 µl of buffer (20 mM Tris-HCl, pH 7.5, 150 mM NaCl, 1 mM EDTA, 1 mM EGTA, 1% Triton X-100, 2.5 mM sodium pyrophosphate, 1 mM Na3VO4, 1 mM NaF, 1 µg/mL leupeptin, 1 µg/mL aprotinin, 1 mM phenylmethylsulfonyl fluoride). Sample lysates were incubated on ice for 10 min and debris was separated by centrifuge; protein concentration in the resulting supernatant was measured using the Pierce BCA Protein Assay Kit (Thermo Fisher Scientific Inc; Waltham, MA, USA). Protein concentrations were normalized and treated with an equal volume of activation mix (50% glycerol, 50 µM ATP, 60 mM MgCl_2_, 0.05% Brij 35, 0.25 mg/mL bovine serum albumin). Samples were spotted onto peptide arrays (JPT Peptide Technologies GmbH, Berlin, Germany) containing annotated kinase phosphosites. Sample slides were incubated for 2 h at 37 °C and 5% CO_2_. Kinome peptide arrays were washed with PBS (Gibco) containing 1% Triton X-100 and rinsed with deionized H_2_O. Arrays were treated with PRO-Q Diamond phosphoprotein stain (Invitrogen, Carlsbad, CA, USA) for 1 h with gentle agitation, followed by three cycles of destain (20% acetonitrile, 50 mM sodium acetate, pH 4.0) for ten minutes each, followed by a final rinse in deionized H_2_O for 10 min. Array slides were tapped against paper towel to remove excess moisture placed in 50-ml conical tubes to centrifuge at 300 × *g* until dry. Peptide-binding signal intensity at each array spot was captured using a PowerScanner microarray scanner (Tecan, Morrisville, NC, USA) with a 580-nm filter and running Array-Pro Analyzer software (version 6.3, Media Cybernetics, Rockville, MD, USA).

### Kinome data processing and analysis

Kinome data was handled as previously described^36^. Briefly, the background signal intensities of peptide arrays were subtracted from foreground signal intensities, and cross-peptide data was normalized using the variance stabilization model^37^. For each peptide, the average of three technical replicate signal intensity values for EBOV trVLP-treated samples was compared against that of time-matched controls; changes in phosphorylation were identified by paired *t* tests. Mathematical analysis of data was performed using the Platform for Integrated, Intelligent Kinome Analysis 2 (PIIKA2) software^38^. Hierarchical clustering of data was performed by comparing all peptides, and then by comparing only peptides that reported consistent results across biological replicates. The Pearson correlation distance metric and the McQuitty linkage method were used for hierarchical clustering. The online tool Metascape (https://metascape.org; accessed October 5, 2023) was used to perform gene ontology analysis using proteins that displayed differential phosphorylation (FC ≥ 1; *P* ≤ 0.05) at one or more phosphosites^30^. Results for gene list queries were limited Reactome Gene Sets (https://reactome.org; accessed October 5, 2023)^31^.

### Trans-epithelial/endothelial electrical resistance testing

To test the effect of EBOV infection on cell barrier function, we used the ECIS Z-Theta system (Applied BioPhysics; Troy, NY, USA) to measure resistance of monolayers of Leydig cells and Sertoli cells to transepithelial electrical current. Cells were seeded on 96W20idf PET plates (Applied BioPhysics) at a concentration of 30,000 cells/well and incubated for 48 h at 37 °C with 5% CO_2_. After incubation, 200 ul of cell media was replaced with 200 ul per well of one of: media collected from HEK293T cells used for EBOV trVLP production, media collected from HEK293T cells used as negative controls for EBOV trVLP production (baseline resistance), or media containing 1% Triton-X100 (barrier disruption). For each cell type, 12 wells were allocated per treatment, with an additional 12 wells remaining unseeded for ECIS standardization purposes; prior to cell treatment, any well displaying technical malfunctions were excluded from the test. Readings were collected continuously for 24 h following treatment, after which media was replaced and readings continued for an additional 24 h. Resistance was considered a measure electrical impedance across cell-cell tight junctions, and electrical capacitance was considered as a measure of electrical impedance across cell lipid bilayers^39,40^. Resistance readings were collected at frequencies ranging from 250 Hz to 6.40 × 10^4^ Hz, to determine the frequency with the least background noise for each set of readings.

## Results

### Leydig cells and Sertoli cells are permissive to infectious EBOV

**Fig. 1:**
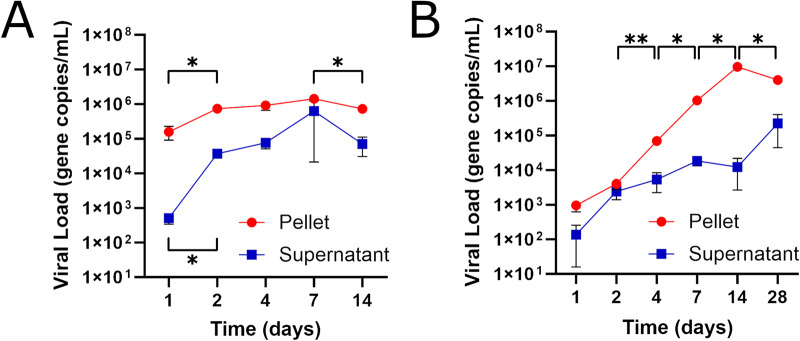
EBOV replication kinetics in Leydig cells and Sertoli cells. Quantitation by RT-PCR of VP40 gene copies in cell pellet and supernatant of Leydig cells (**A**) and Sertoli cells (**B**) treated with infectious EBOV. Primer sequences for strand-specific RNA reverse transcription and quantitation of the VP40 gene are listed in Table 1. The cutoff for minimum threshold of detection was at 36 cycles. Each data point represents the average of three biological replicates, for each of which two technical replicates were performed during quantitation. For each cell type, significant changes in viral load between neighboring time points were determined using the Student’s *t* test. Asterisks indicate a significant change in viral load, where *, **, ***, and **** indicates that P ≤ 0.05, P ≤ 0.01, P ≤ 0.001, and P ≤ 0.0001, respectively.

We used infectious EBOV to test viral tropism and replication kinetics in mouse Leydig cells and Sertoli cells. Beginning 1 dpi, we detected increases in EBOV in cell pellet and supernatant of Leydig cell (Fig. 1A) and Sertoli cell (Fig. 1B) tissue cultures. The intracellular viral load of Leydig cells was more than 100-fold that of Sertoli cells 1 dpi, and only Leydig cells displayed increases (*P* ≤ 0.05) in intracellular and extracellular EBOV between 1 dpi and 2 dpi. Starting 2 dpi, viral load in Sertoli cells increased (*P* ≤ 0.05) before peaking 14 dpi at quantities approximately 10-fold those of Leydig cells. Infectious EBOV-treated and mock-treated Leydig cell tissue cultures lost viability around 14 dpi, while infectious EBOV-treated and mock-treated Sertoli cells remained viable past 28 dpi.

### Differential gene expression of Leydig and Sertoli cells in response to infectious EBOV

**Fig. 2:**
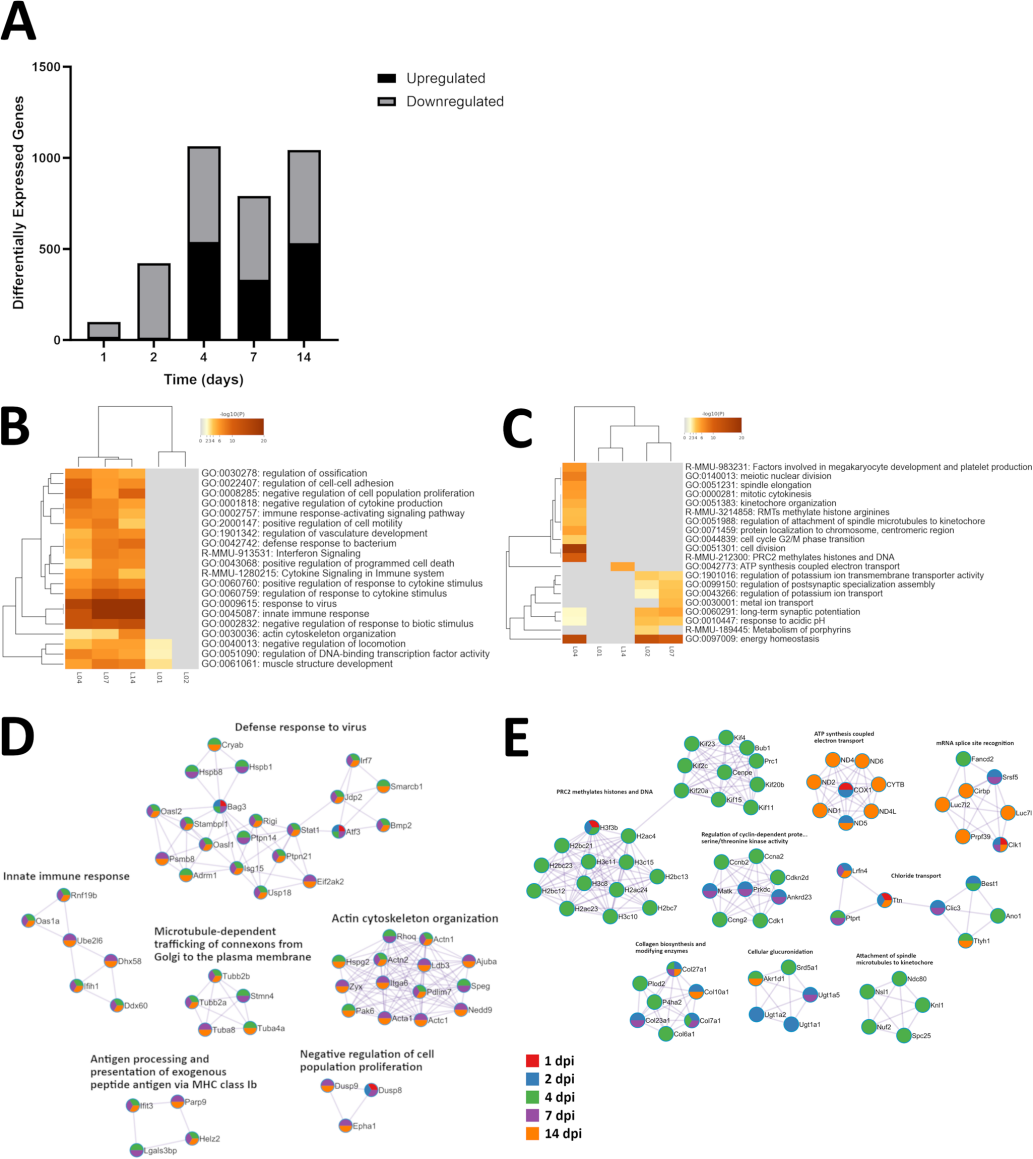
Cluster analysis of differentially expressed genes in EBOV-infected Leydig cells. **A** Counts of significantly upregulated (log2 FC ≥ 2; P ≤ 0.05) and downregulated (log2 FC ≤ −2; P ≤ 0.05) genes for Leydig cells. Values were calculated from three replicates for each set of variables, using normalized feature counts determined by RNAseq analysis. For each time point, separate queries of upregulated and downregulated genes were performed against the Gene Ontology Biological Processes (https://geneontology.org; accessed October 5, 2023) and Reactome Gene Sets (https://reactome.org; accessed October 5, 2023) to identify associated ontological terms^31–34^. Significance of associated terms was determined by calculating accumulative hypergeometric *p*-values (cutoff: 0.01) and enrichment factors (minimum 1.5). Samples were clustered based by significant terms using Kappa-statistical similarities (0.3 kappa threshold) among upregulated (**B**) and downregulated (**C**) member genes. A heatmap was generated and colored by *p*-values, where white cells indicate the lack of enrichment for that term in the corresponding gene list. The term with the best *p*-value for each term cluster was used as a representative on the heatmap. Protein-protein interaction networks were generated based on upregulated (**D**) and downregulated (**E**) genes, and the MCODE algorithm was applied to these networks to identify neighborhoods where proteins are densely connected. Enrichment analysis was applied to each MCODE network to extract “biological meanings” from the network component, where top three best *p*-value terms were retained and represented based by one term per cluster as a label. A breakdown of all MCODE interpretations for each time point is available as supplementary data. Nodes represent individual proteins, node pie sectors indicate which samples differentially expressed each protein, and edges represent interactions between proteins. Analysis was performed using the Metascape online tool (https://metascape.org; accessed October 5, 2023), which incorporates network visualization by Cytoscape^30,71^. L01, Leydig + infectious EBOV 1 dpi; L02, Leydig + infectious EBOV 2 dpi; L04, Leydig + infectious EBOV 4 dpi; L07, Leydig + infectious EBOV 7 dpi; L14, Leydig + infectious EBOV 14 dpi.

The number genes in EBOV-infected Leydig cells that were differentially expressed (i.e. significantly upregulated or downregulated) compared to control cells increased over the first 4 dpi (Fig. 2A). A lack of upregulated genes during initial EBOV infection limited associations with ontologies for samples collected 1 dpi and prevented association for samples collected 2 dpi (Fig. 2B). Samples collected 4, 7, and 14 dpi clustered together based on upregulation of genes across highly enriched ontologies. A lack of downregulated genes also hindered ontological clustering for samples collected 1 dpi, and to a lesser extent for samples collected 14 dpi (Fig. 2C). In this case, samples collected 2 dpi and 7 dpi were grouped together, and a clear divergence in differential gene expression caused samples collect 4 dpi to cluster alone, despite several ontological terms being shared with samples from other time points. Overall, upregulated genes were most associated with pathways for innate immune response, response to virus, and regulation of response to biological stimulus; downregulated genes were most associated with pathways for nuclear chromosome segregation, mitotic cell cycle progress, and mitotic cell cycle (Table 2). These associations appeared to be heavily influenced by data from later time points, whereas differential gene expression was primarily associated with the cell membrane and extracellular matrix. When ontological clustering was considered in terms of protein-protein interactions, samples collected 4, 7, and 14 dpi often upregulated the same genes for ontological terms (Fig. 2D). In comparison, downregulated genes were rarely shared by samples collected at different time points (Fig. 2E).

**Table 2:** Biological meaning of differential gene expression in Leydig cells infected with EBOV relative to mock-infected controls

Leydig	Upregulated	Downregulated
Overall	GO:0045087|innate immune response|−25.6	GO:0098813|nuclear chromosome segregation|−17.7
	GO:0009615|response to virus|−19.9	GO:1903047|mitotic cell cycle process|−16.9
	GO:0002831|regulation of response to biotic stimulus|−18.3	GO:0000278|mitotic cell cycle|−16.2
1 dpi	GO:0198738|cell-cell signaling by wnt|−3.7	No significant terms
	GO:0016055|Wnt signaling pathway|−3.7	
	GO:0007517|muscle organ development|−3.6	
2 dpi	No significant terms	R-MMU-1474244|Extracellular matrix organization|−5.7
		R-MMU-8948216|Collagen chain trimerization|−5.1
		GO:0052695|cellular glucuronidation|−4.4
4 dpi	GO:0002831|regulation of response to biotic stimulus|−15.1	GO:0051301|cell division|−18.3
	GO:0045088|regulation of innate immune response|−13.7	GO:0098813|nuclear chromosome segregation|−16.7
	GO:0009615|response to virus|−13.5	GO:0000278|mitotic cell cycle|−15.4
7 dpi	GO:0045087|innate immune response|−21.7	GO:0051000|positive regulation of nitric-oxide synthase activity|−4.1
	GO:0009615|response to virus|−18.7	GO:0060485|mesenchyme development|−3.7
	GO:0045088|regulation of innate immune response|−15.7	GO:0099150|regulation of postsynaptic specialization assembly|−3.6
14 dpi	GO:0045087|innate immune response|−23.6	GO:0042773|ATP synthesis coupled electron transport|−8.7
	GO:0009615|response to virus|−19.5	GO:0019646|aerobic electron transport chain|−7.9
	GO:0002831|regulation of response to biotic stimulus|−17.9	GO:0022904|respiratory electron transport chain|−7.8

The top MCODE result, composed of the top three terms within that MCODE, is listed for upregulated and downregulated genes overall and at each time point. The databased ID and *p*-value for each term is also provided.

**Fig. 3:**
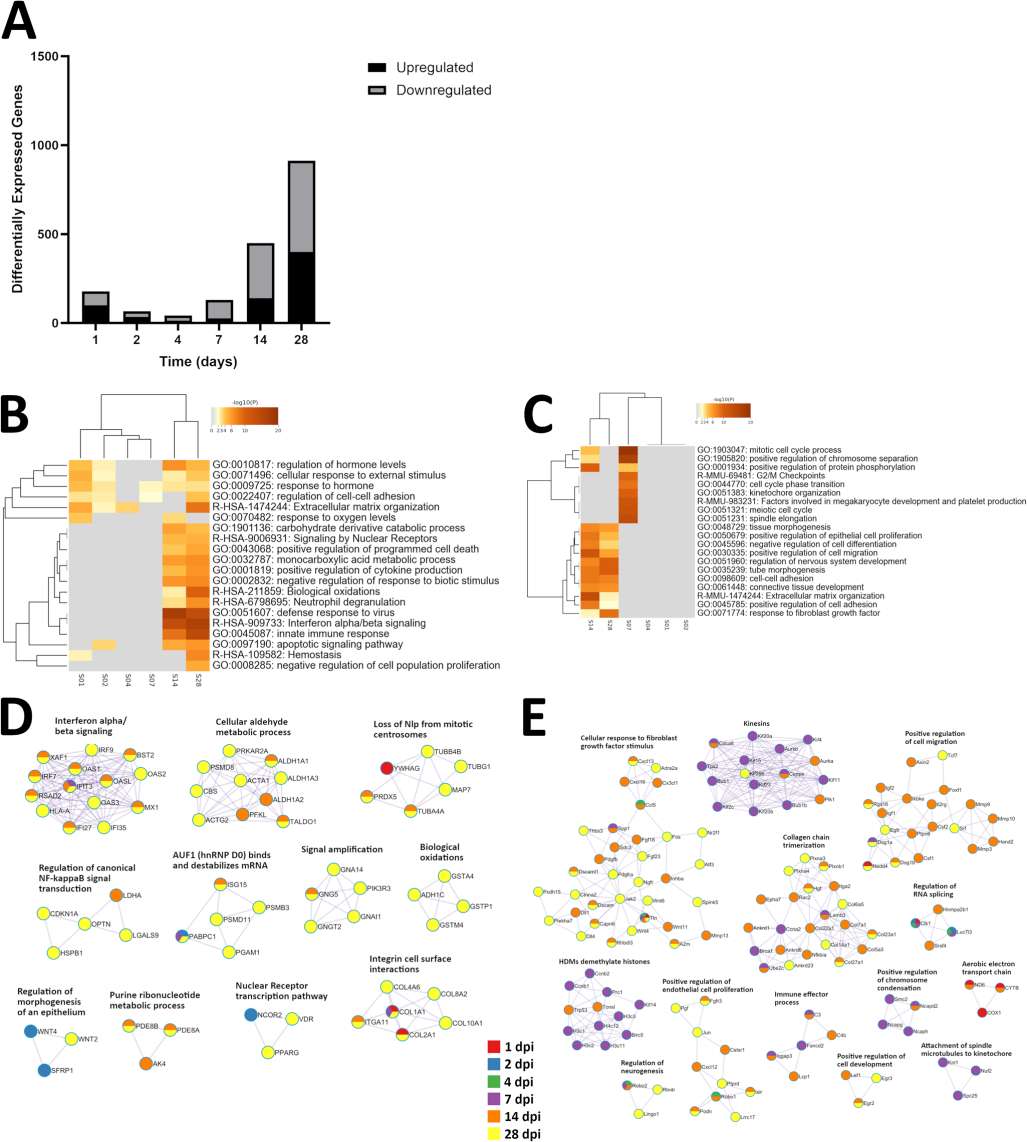
Cluster analysis of differentially expressed genes in EBOV-infected Sertoli cells compared to mock-infected cells. **A** Counts of significantly upregulated (log2 FC ≥ 2; *P* ≤ 0.05) and downregulated (log2 FC ≤ −2; *P* ≤ 0.05) genes for Sertoli cells. Values were calculated from three replicates for each set of variables, using normalized feature counts determined by RNAseq analysis. For each time point, separate queries of upregulated and downregulated genes were performed against the Gene Ontology Biological Processes (https://geneontology.org; accessed October 5, 2023) and Reactome Gene Sets (https://reactome.org; accessed October 5, 2023) to identify associated ontological terms. Significance of associated terms was determined by calculating accumulative hypergeometric *p*-values (cutoff: 0.01) and enrichment factors (minimum 1.5)^31–34^. Samples were clustered based by significant terms using Kappa-statistical similarities (0.3 kappa threshold) among upregulated (**B**) and downregulated (**C**) member genes. A heatmap was generated and colored by *p*-values, where white cells indicate the lack of enrichment for that term in the corresponding gene list. The term with the best *p*-value for each term cluster was used as a representative on the heatmap. Protein-protein interaction networks were generated based on upregulated (**D**) and downregulated (**E**) genes, and the MCODE algorithm was applied to these networks to identify neighborhoods where proteins are densely connected. Enrichment analysis was applied to each MCODE network to extract “biological meanings” from the network component, where top three best *p*-value terms were retained and represented based by one term per cluster as a label. A breakdown of all MCODE interpretations for each time point is available as supplementary data. Nodes represent individual proteins, node pie sectors indicate which samples differentially expressed each protein, and edges represent interactions between proteins. Analysis was performed using the Metascape online tool (https://metascape.org; accessed October 5, 2023), which incorporates network visualization by Cytoscape^30,71^. S01, Sertoli + infectious EBOV 1 dpi; S02, Sertoli + infectious EBOV 2 dpi; S04, Sertoli + infectious EBOV 4 dpi; S07, Sertoli + infectious EBOV 7 dpi; S14, Sertoli + infectious EBOV 14 dpi; S28, Sertoli + infectious EBOV 28 dpi.

The number of genes differentially expressed in EBOV-infected Sertoli cells compared to control cells decreased over the first 4 days post-infection (Fig. 3A). Sertoli samples clustered sequentially based on upregulated genes, although in the case of samples collected 4 dpi and 7 dpi this was largely due to a lack of differential gene expression rather than shared upregulation (Fig. 3B). Samples collected 1 dpi and 2 dpi shared most of the ontological terms they possessed with samples collected 14 dpi and 28 dpi, although the later time points inhabited a separate cluster due to a much broader range of enriched ontological terms. Samples collected 4 dpi and 7 dpi associated with few ontological terms, primarily due to low numbers of differentially expressed genes. In a similar manner, the lack of downregulated genes for samples collected 1, 2, and 4 dpi hindered their association with ontological terms (Fig. 3C). Samples collected 7 dpi did not cluster with those of other time points due to several unique ontological terms, while samples collected 14 dpi and 28 dpi again clustered together based on many shared ontologies. Overall, upregulated genes were most associated with pathways for defense response to virus, defense response to symbiont, and response to virus; downregulated genes were most associated with mitotic cell cycle process, mitotic cell cycle, and nuclear chromosome segregation (Table 3). Of note was the distinct association of samples collected 1 dpi with energy production, and of samples collect 2 dpi with steroid signaling. Analysis of protein-protein interactions indicated that only samples collected 14 dpi and 28 dpi shared a majority of upregulated genes in enriched pathways, and more specifically those for antiviral response and ribonucleotide metabolism (Fig. 3D). In comparison downregulated genes in enriched pathways tended to be unique to samples collected at one time point (Fig. 3E).

**Table 3:** Biological meaning of differential gene expression in Sertoli cells infected with EBOV relative to mock-infected controls

Sertoli	Upregulated	Downregulated
Overall	GO:0051607|defense response to virus|−21.2	GO:1903047|mitotic cell cycle process|−27.1
	GO:0140546|defense response to symbiont|−21.1	GO:0000278|mitotic cell cycle|−26.4
	GO:0009615|response to virus|−20.6	GO:0098813|nuclear chromosome segregation|−24.1
1 dpi	GO:0031667|response to nutrient levels|−6.3	GO:0019646|aerobic electron transport chain|−5.2
	GO:0009991|response to extracellular stimulus|−6.1	GO:0042775|mitochondrial ATP synthesis coupled electron transport|−5.0
	R-HSA-9006934|Signaling by Receptor Tyrosine Kinases|−5.9	GO:0042773|ATP synthesis coupled electron transport|−4.9
2 dpi	GO:0033143|regulation of intracellular steroid hormone receptor signaling pathway|−5.1	No significant terms
	GO:0030879|mammary gland development|−4.3	
	GO:0008584|male gonad development|−4.2	
4 dpi	No significant terms	No significant terms
7 dpi	No significant terms	GO:1903047|mitotic cell cycle process|−46.0
		GO:0000278|mitotic cell cycle|−45.4
		GO:0051301|cell division|−43.6
14 dpi	GO:0051607|defense response to virus|−20.8	R-MMU-1474244|Extracellular matrix organization|−16.1
	GO:0140546|defense response to symbiont|−20.8	GO:0030335|positive regulation of cell migration|−13.2
	GO:0009615|response to virus|−18.2	GO:0040017|positive regulation of locomotion|−13.2
28 dpi	R-HSA-909733|Interferon alpha/beta signaling|−18.9	GO:0035239|tube morphogenesis|−13.3
	GO:0045087|innate immune response|−15.8	GO:0051960|regulation of nervous system development|−12.8
	R-HSA-913531|Interferon Signaling|−15.7	GO:0001944|vasculature development|−11.7

The top MCODE result, composed of the top three terms within that MCODE, is listed for upregulated and downregulated genes overall and at each time point. The databased ID and *p*-value for each term is also provided.

### Leydig cells and Sertoli cells are permissive to EBOV trVLP entry and transcription

**Fig. 4:**
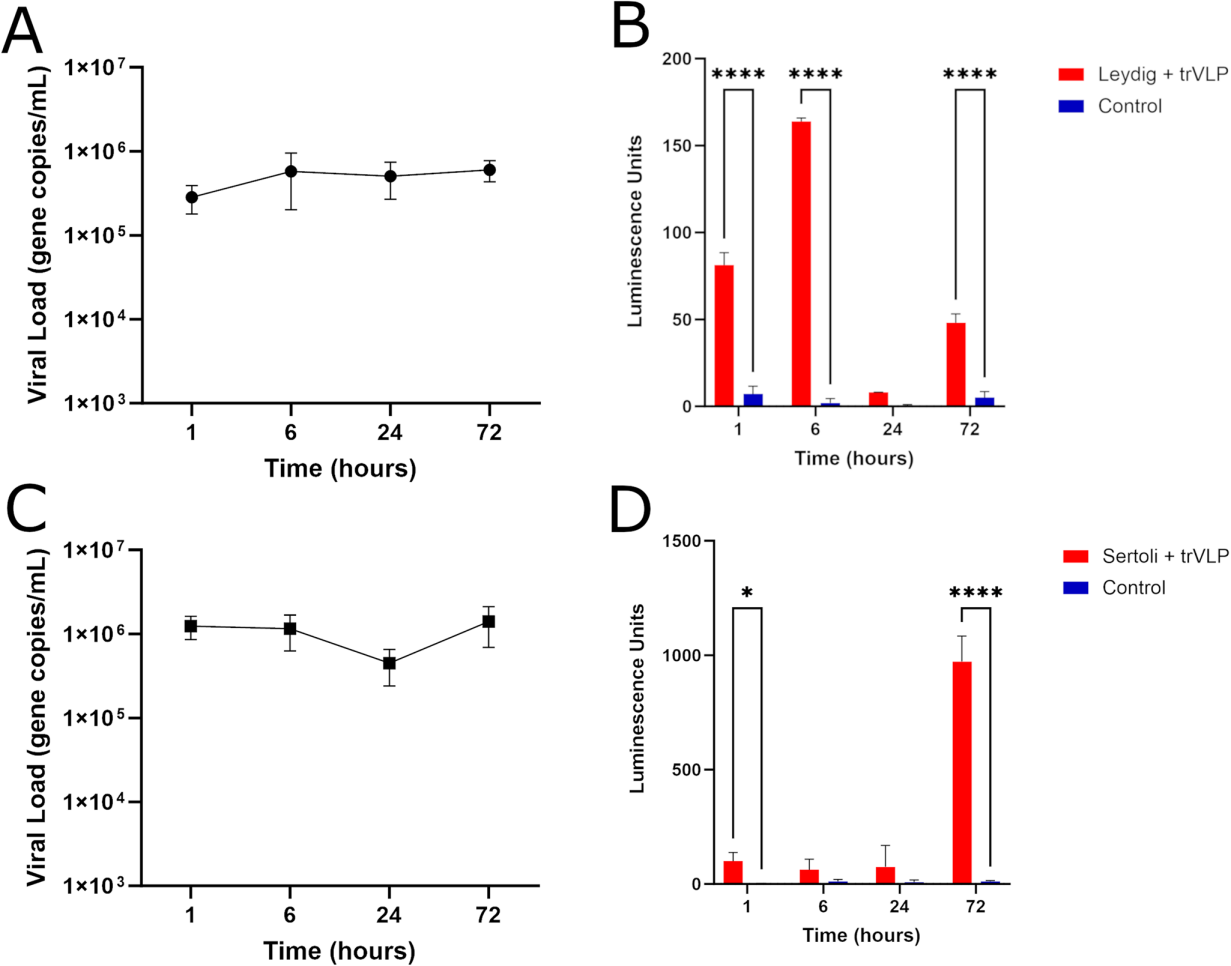
Leydig cells and Sertoli cells are permissive to trVLP entry and transcription. **A** Quantification of VP40 gene copies in Leydig cells treated with EBOV trVLP. **B** Luminescence of the firefly luciferase reporter in mock-treated and EBOV trVLP-treated Leydig cells. **C** Quantitation of VP40 gene copies in Sertoli cells treated with EBOV trVLP. **D** Luminescence of the firefly luciferase reporter in mock-treated and EBOV trVLP-treated Sertoli cells. For each time point, EBOV VP40 RNA and luciferase luminescence was quantified based on three biological replicates and two technical replicates. Primer sequences for strand-specific RNA reverse transcription and quantitation of viral VP40 are listed in Table 1. Significant differences in firefly luciferase luminescence between mock-treated and EBOV trVLP-treated cells was determined using 2-way ANOVA. Significant changes in quantity of EBOV VP40 between neighboring time points were determined using the Student’s *t* test. Asterisks indicate a significant difference, where *, **, ***, and **** indicates that *P* ≤ 0.05, *P* ≤ 0.01, *P* ≤ 0.001, and *P* ≤ 0.0001, respectively.

Media collected from EBOV trVLP-producing HEK293T cells was determined to contain approximately 2.59 × 10^8^ EBOV VP40 gene copies per ml. The presence of EBOV VP40 gene copies attached to or within Leydig cells was observed as early as 1 h (Fig. 4A). In addition, significant increases in reporter luminescence in EBOV trVLP-treated Leydig cells compared to mock-treated Leydig cells was noted at 1 h (*P* ≤ 0.0001) and 6 h (*P* ≤ 0.0001) but not 24 h post treatment (Fig. 4B). Sertoli cells also displayed evidence of interaction with EBOV trVLP within 1 h of treatment, and a significant difference in reporter luminescence between EBOV trVLP-treated cells and mock-treated cell was observed 1 h post treatment (≤0.05) but not at 6 h or 24 h (Fig. 4C and D, respectively). After 24 h, treatment media was replaced with fresh media and 48 h later both cell lines displayed significant (*P* ≤ 0.0001) differences in luminescence between EBOV trVLP-treated and mock-treated samples.

### Early signaling activity is inhibited in Leydig cells, but not in Sertoli cells

**Fig. 5:**
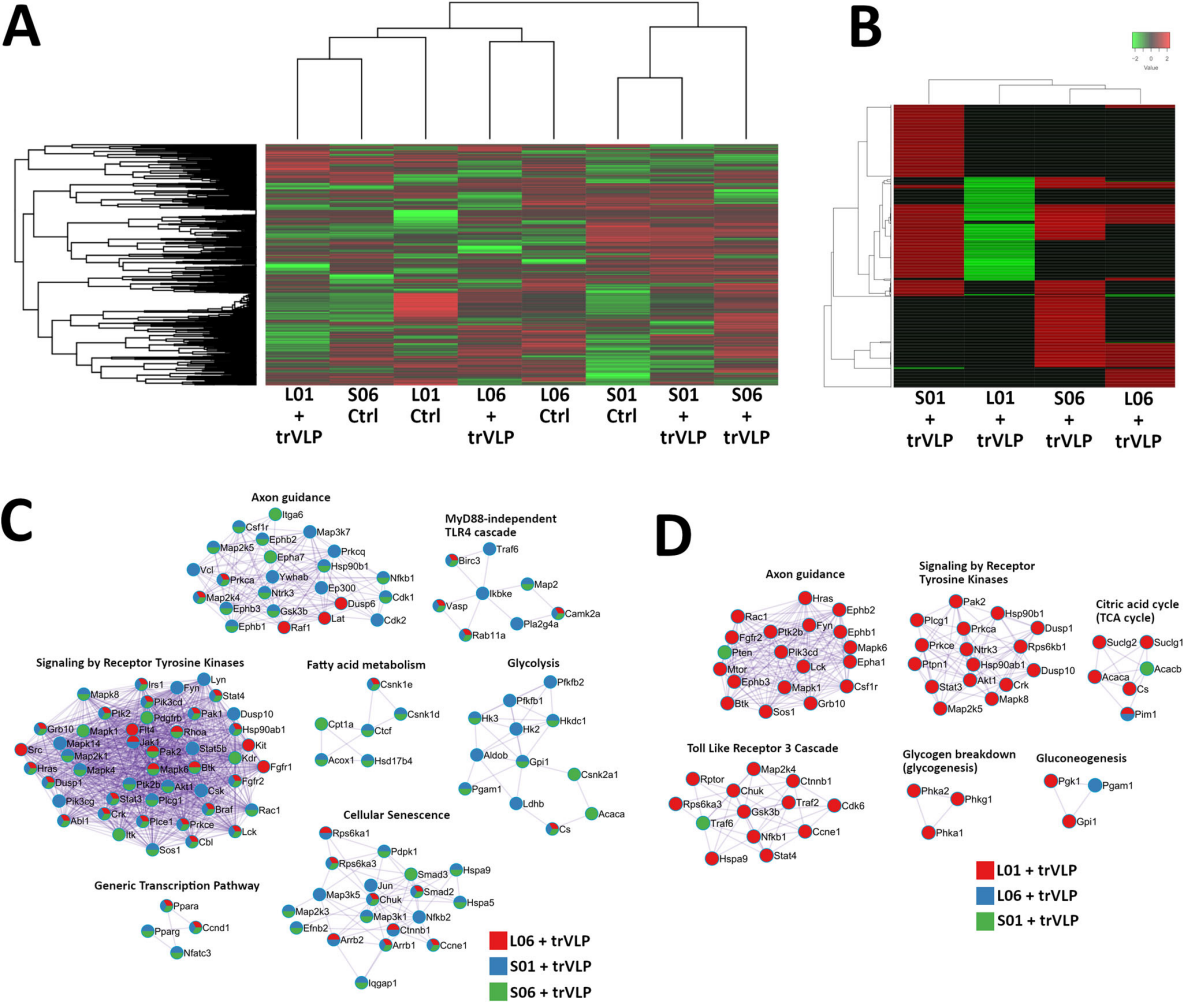
Assessment of kinome response differences between Leydig cells and Sertoli cells treated with EBOV trVLP. For each peptide, the average of three technical replicate signal intensity values for EBOV trVLP-treated samples was compared against that of time-matched controls; changes in phosphorylation were identified by paired *t* tests. Mathematical analysis of data was performed using the Platform for Integrated, Intelligent Kinome Analysis 2 (PIIKA2) software^38^. The Pearson correlation distance metric and the McQuitty linkage method were used for hierarchical clustering to compare phosphorylation at all peptides (**A**), and then by comparing only peptides that reported consistent results across biological replicates (**B**). Protein-protein interaction networks were generated based on increased (**C**) and decreased (**D**) phosphorylation of peptides. Ontological terms were assigned based on Reactome Gene Sets (https://reactome.org; accessed October 5, 2023) and the MCODE algorithm was applied to these networks to identify neighborhoods where proteins are densely connected^31^. Enrichment analysis was applied to each MCODE network to extract “biological meanings” from the network component, where top three best *p*-value terms were retained and represented by one term per cluster as a label. Nodes represent individual proteins, node pie sectors indicate which samples differentially expressed each protein, and edges represent interactions between proteins. Protein-protein interaction analysis was performed using the Metascape online tool (https://metascape.org; accessed October 5, 2023), which incorporates network visualization by Cytoscape^30,71^. L01, Leydig cells 1 h post treatment; L06, Leydig cells 6 h post treatment; S01, Sertoli cells 1 h post treatment; S06, Sertoli cells 6 h post treatment.

We tested the effects of EBOV trVLP on signaling activity of Leydig cells and Sertoli cells during early infection by examining kinase phosphorylation activity at individual phosphosites. Hierarchical clustering of samples by peptide phosphorylation reported preferential grouping by cell line rather than treatment (Fig. 5A). When only significant changes in peptide phosphorylation were compared, and after mock-infected control data sets were subtracted, Leydig cells at 1 h post treatment were notable for their comprehensive reduction in signaling activity (Fig. 5B). Enrichment analysis revealed that increased phosphorylation of proteins associated with axon guidance, fatty acid metabolism, and glycolysis was primarily limited to Sertoli cells 1 h and 6 h post treatment (Fig. 5C). Ontological terms such as Myd88-independent Tlr4 cascade, cellular senescence, and signaling by receptor tyrosine kinases were shared by Leydig cells 6 h post treatment and both Sertoli cell samples. The lack of increased phosphorylation observed in Leydig cells 1 h post treatment prevented its inclusion in this analysis, while the lack of decreased phosphorylation observed in the other samples prevented their inclusion in the complementary ontological enrichment analysis (Fig. 5D). Primarily driven by Leydig cells 1 h post treatment, ontological terms associated with decreased phosphorylation included axon guidance, toll like receptor cascade, and signaling by receptor tyrosine kinases. We also interpreted the biological meaning of protein-protein interactions based on one or more significantly changed phosphorylation site. All samples with increased phosphorylation data were primarily associated with signaling by receptor tyrosine kinases and cytokine signaling in the immune system; decreased phosphorylation in Leydig cells 1 h post treatment was primarily associated with signaling by receptor tyrosine kinases, cytokine signaling in the immune system, and signaling by interleukins (Table 4).

**Table 4:** Biological meaning of increased and decreased phosphorylation activity in Leydig cells and Sertoli cells treated with EBOV trVLP relative to untreated controls

Increased phosphorylation	
Leydig 6 h post treatment	R-MMU-9006934|Signaling by Receptor Tyrosine Kinases|−26.8
	R-MMU-5683057|MAPK family signaling cascades|−14.2
	R-MMU-1280215|Cytokine Signaling in Immune system|−13.9
Sertoli 1 h post treatment	R-MMU-1280215|Cytokine Signaling in Immune system|−28.1
	R-MMU-9006934|Signaling by Receptor Tyrosine Kinases|−28.0
	R-MMU-449147|Signaling by Interleukins|−25.8
Sertoli 6 h post treatment	R-MMU-9006934|Signaling by Receptor Tyrosine Kinases|−26.2
	R-MMU-1280215|Cytokine Signaling in Immune system|−21.3
	R-MMU-422475|Axon guidance|−20.4
**Decreased phosphorylation**	
Leydig 1 h post treatment	R-MMU-9006934|Signaling by Receptor Tyrosine Kinases|−21.4
	R-MMU-1280215|Cytokine Signaling in Immune system|−20.0
	R-MMU-449147|Signaling by Interleukins|−16.9

The top MCODE result, composed of the top three terms within that MCODE, is listed for each cell line and time point. The databased ID and *p*-value for each term is also provided. Samples for Sertoli cells 1 h and 6 h post treatment, and for Leydig cells 6 h post treatment contained too few instances of decreased phosphorylation to draw conclusions, while Leydig cells 1 h post treatment contained too few instances of increased phosphorylation.

### Analysis of Leydig and Sertoli cell membrane integrity by ECIS

**Fig. 6:**
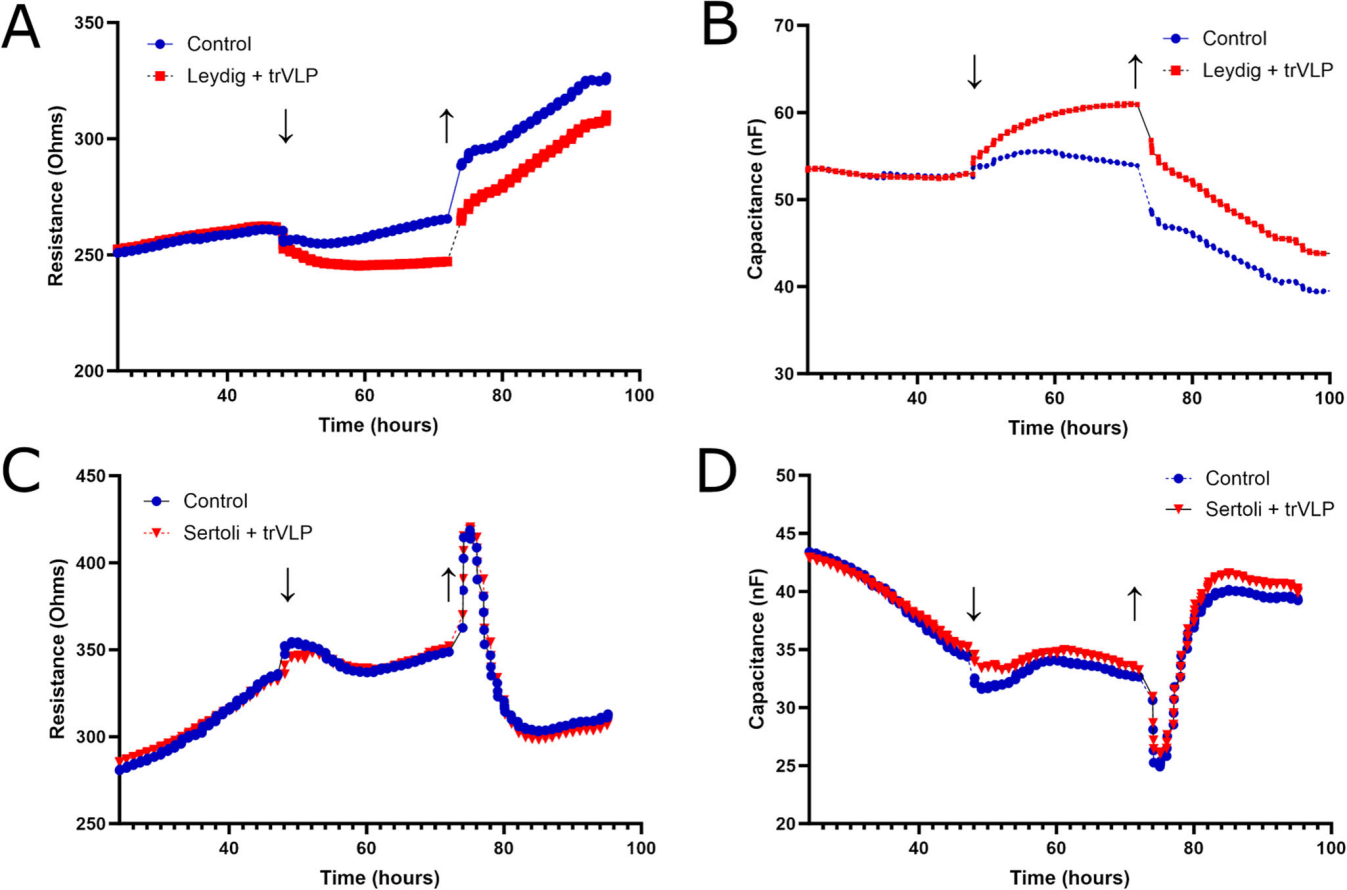
Characterization of longitudinal cell membrane integrity in Leydig and Sertoli cells following EBOV trVLP treatment. Cells were seeded 48 h prior to treatment, and the figures in question display membrane resistance (**A**, **C**) and cell capacitance (**B**, **D**) from 24 h to 96 h after seeding. Data points represent the average value of nine wells per treatment per cell line, as three of the original twelve columns were removed from the experiment prior to treatment due to failed quality control readings. The point at which treatments were added is represented by a downward-pointing arrow (↓), and the point at which media was replaced is indicated by an upward-pointing arrow (↑). Readings were paused while the ECIS plate was manipulated to apply treatments replace media. Resistance readings are displayed at 8.00 × 10^3^ Hz and capacitance readings are displayed at 6.40 × 10^4^ Hz.

In order to determine the effect of EBOV binding and entry BTB function, we treated Leydig cell and Sertoli cell monolayers with EBOV trVLP and measured trans-epithelial/endothelial electrical resistance over time. Leydig cells treated with EBOV trVLP exhibited a decrease in membrane resistance that had begun to resolve by 96 h after media containing EBOV trVLP had been replaced with fresh media (Fig. 6A). Significant differences (*p* < 0.05) were found between the two conditions from 57 to 78 h. There was a corresponding increase in capacitance of Leydig cells treated with EBOV trVLP with significant differences (p < 0.05) between the conditions from 58 h onwards that also appeared to be stabilizing by 96 h after media had been replaced (Fig. 6B). At the same time, electrical resistance of Sertoli cells was unaffected in the presence of EBOV trVLP for 24 h, although limited differences between treatments were noted following replacement of media at 72 h (Fig. 6C). Sertoli cell capacitance was more greatly affected than resistance, with a more notable increase in capacitance occurring in EBOV trVLP-treated cells after 72 h and significant differences (*p* < 0.05) between the conditions from 83 to 95 h (Fig. 6D).

## Discussion

Our objective was to elucidate the interactions between host and viral factors that facilitate persistent EBOV infection of the male reproductive tract. To do this, we treated Leydig cells and Sertoli cells with infectious EBOV and EBOV trVLP. Both cell types were permissive to infectious EBOV, which is consistent with previous studies that reported viral material in the interstitial space and/or the BTB of the testis in EBOV-infected monkeys^14–16^. We observed a stark difference in early intracellular viral load for Leydig cells and Sertoli cells, which could suggest greater efficiency of entry in Leydig cells, but our experiments with EBOV trVLP were less conclusive. Interactions between cell lines and EBOV trVLP were observed within 1 h of treatment, but early viral transcription appeared to occur over a longer period in Leydig cells compared to Sertoli cells. The difference in transcription activity between cell lines may be related to alternate mechanisms of endocytosis and processing that are available to Sertoli cells but not Leydig cells. EBOV enters cells using mechanisms for uptake of phosphatidylserine-presenting cellular debris and cholesterol-dependent fusion with the endosomal membrane. Sertoli cells act as amateur phagocytes to clear apoptotic germ cells from the seminiferous tubules and to collect cholesterol for use during spermatogenesis^23,41^. As such, Sertoli cells possess a distinct phosphatidylserine class B scavenger receptor type I (SR-B1) responsible for selective uptake of cholesterol derived from high-density lipoprotein and apoptotic germ cells^23,42^. Intracellular processing of components from apoptotic germ cells requires intensive regulation of endocytosis and signaling activity, which could affect the timing and localization of EBOV trVLP release into the cytosol^23,41^. Uptake of EBOV by phagocytosis may also factor into early targeting of macrophages and dendritic cells during EBOV infection, which occurs for some other viruses^43^. The apparent lack of EBOV trVLP transcription activity in Sertoli cells 6 h post treatment could also be indicative of the rapid and robust signaling response that we observed in Sertoli cells using kinome arrays.

We also found that early host cell signaling in response to EBOV is limited in Leydig cells compared to Sertoli cells. Kinase phosphorylation was inhibited in Leydig cells 1 h after treatment with EBOV trVLP, while Sertoli cells showed a robust increase in signaling activity. Furthermore, while phosphorylation increased in both cell lines 6 h after treatment, the magnitude of increase in Leydig cells was still limited compared to Sertoli cells. Sertoli cells limit the immune system at the BTB, but this is offset by a broad range of pattern recognition receptors for defense^44^. We propose that after EBOV enters Sertoli cells they detect and respond to the virus rapidly enough to limit further viral entry and transcription. Our transcriptomic analysis of EBOV-infected cells supports this conclusion; a greater number of genes were differentially expressed in Sertoli cells infected with EBOV 1 dpi compared to Leydig cells, and part of the response by Sertoli cells was upregulation of host signaling and membrane function pathways. This response by Sertoli cells may be indicative of attempts by host cells to maintain homeostasis while EBOV induces cell replication activity and alters membrane permeability as an infection strategy^45–47^. Dysregulation of normal activity at the cell membrane and extracellular matrix can facilitate pathogenic invasion and disruption of cell-cell signaling, as may be the case here^48,49^. When we measured changes in trans-epithelial/endothelial electrical resistance of cell monolayers treated with EBOV trVLP we observed limited decreases in membrane resistance and increases in capacitance for Leydig cells. In comparison, the impact of EBOV trVLP on Sertoli cell membrane resistance and capacitance was less apparent. If electrical resistance values represent tight-junction function and capacitance represents impedance of electricity across cell lipid bilayers, then Sertoli cells appear to be more proficient at maintaining tissue barrier and cell membrane integrity in response to EBOV^39,40^. Disassembly and reassembly of the various Sertoli cell-cell junctions during spermatogenesis is reliant on tightly regulated cholesterol signaling associated with endocytosis, endosome-mediated transcytosis, recycling, and endosome-mediated degradation^41,50^. The stepwise and time-sensitive nature of spermatogenesis necessitates rapid detection and response to changes in prevalence and composition of signaling and transport factors at the plasma membrane, and as such Sertoli cells may be better equipped to counteract dysregulation induced by EBOV^51^. Regardless, the differences we observed between cell lines in response to EBOV binding and entry should be investigated further, as the disparity in intracellular viral load 1 dpi likely affected changes in gene expression of each cell line in response to viral transcription and replication over time.

Early Leydig cell transcriptomic response data from our investigation suggested that this cell type was more conducive to EBOV infection. Early upregulation of genes related to WNT signaling was notable in Leydig cells; WNT/β-catenin signaling increases expression of cyclin D1, which in turn promotes cell cycle progression^52^. Further, EBOV VP40 has been shown to increase expression of cyclin D1, and cell cycle inhibitors can hinder EBOV infection^45,46^. In comparison, Sertoli cells prioritized increases in expression of genes related to detection of, and response to, the extracellular environment, alongside a decrease in expression of genes for energy production. Increased prioritization of detection and response mechanisms supports our kinome analysis of Sertoli cells treated with EBOV trVLP, while decreases in energy production may be indicative of Sertoli cells counteracting induction of cell cycle activity by EBOV. In a previous transcriptomic study, one response of lymphoid tissue infected with EBOV was to decrease expression of genes associated with cell cycle^53^. A modest increase in immune signaling by Sertoli cells was also observed 2 dpi; this is notable as an isolated incident for either cell line during early time points. Previous studies of EVD pathogenesis in humans and animal models have shown that greater survival is linked to an early immune response to EBOV infection, while greater lethality is linked to a strong but delayed immune response, particularly that of the adaptive immune response^54,55^. The earlier response by Sertoli cells may be due in part to more rapid detection of EBOV during endocytosis. It is also possible that the lower initial EBOV viral load in Sertoli cells compared to Leydig cells resulted in less comprehensive interference of host immune response by viral proteins. EBOV proteins are strong inhibitors of interferon signaling pathways, the result of which is that the host antiviral response is delayed while EBOV replication gets underway^56,57^. If the quantity of EBOV proteins was insufficient to completely suppress host immune factors, then the limited immune response observed in Sertoli cells 2 dpi may have served to further limit the EBOV activity. Finally, Sertoli cells upregulated gene expression associated with steroid/hormone signaling 2 dpi. Aside from the association with spermatogenesis, this is notable because emerging evidence suggests that EBOV induces changes in cell metabolism, particularly with regards to fatty acids, steroids, and amino acids^58,59^. After phosphatidylserine-dependent phagocytosis of apoptotic germ cells, Sertoli cells can metabolize component fatty acids and amino acids as oxidation substrates, and lipid droplets are known to form as part of phagocytosis of apoptotic germ cells^60,61^. Further study could elucidate the effects that lipid metabolism and storage have on the EBOV replication cycle.

Our analysis of host transcriptomic responses to EBOV identified a number of potentially universal pathways, albeit with different time frames. As viral load in Sertoli cells approached and passed that of Leydig cells, we noted increases in antiviral response and decreases in cell cycle activity in Sertoli cells that aligned somewhat with those observed in Leydig cells at earlier time points. In addition, Leydig cells and Sertoli cells responded to increasing intracellular and/or extracellular viral loads by increasing gene expression associated with immune response, and by decreasing expression of genes associated with cell cycle activity. From 4 dpi to 14 dpi, Leydig cells displayed sustained upregulation of pathways related to immune response. Sertoli cells did not display a similar response until 14 dpi, and this tended to occur with fewer pathways at lesser magnitudes. In a previous animal study, EBOV-infected Macaque liver and adrenal tissues displayed transcription responses similar to Leydig cells, with late but strong increases in immune response^53^. Leydig cells also decreased expression of genes associated with membrane function, whereas decreases in cell locomotion and migration in Sertoli cells could be interpreted as reinforcement of membrane function. Conflicting signaling of hemostasis activity was observed in both cell lines and may be linked to hemorrhagic factors traditionally associated with EVD^62^. In this case, extended upregulation genes associated with hemostasis may be a response mounted by host cells in opposition to downregulation induced by EBOV. Dysregulation in hemostasis function could explain hemorrhaging that is reported in EVD as endothelial cells undergo coagulative necrosis^63,64^. Finally, Sertoli cells showed a modified immune response and increased priority on cell detoxification towards later stages of infection. It is likely that EBOV induces generation of reactive oxygen species, and fatal cases of EVD report levels of reactive oxygen species higher than those of survivors^53,65,66^. Sertoli cells maintain a complex relationship with reactive oxygen species, which play a role in spermatogenesis, and thus they may be better equipped to handle the cytotoxic effects^67^. When late-stage upregulation of gene associated with reactive oxygen species based lysosomal/phagosomal activity is also considered, it is possible that Sertoli cells compensate for reduced inflammatory signaling by repurposing otherwise-cytotoxic materials for antiviral activity^44,68^.

In the current study, we used infectious EBOV and EBOV trVLP to characterize cellular tropism, viral replication kinetics, and host response in cells of the BTB. Our findings indicate that Sertoli cells respond rapidly to EBOV infection to hinder further viral entry and transcription in a manner that is linked to hormone signaling. This in turn limits dysregulation of host cell signaling and membrane function induced at high viral loads. An unspecified critical viral load appeared to trigger changes in gene expression for innate immune and cell cycle signaling in Leydig cells and Sertoli cells, after which they diverged in areas other than immune response. At late stages of infection, Leydig cells appeared to suffer losses in barrier adhesion, while Sertoli cells actively countered cell motility activity. We showed evidence for persistence of EBOV in Sertoli cells in a manner that did not appear to be inflammatory or compromise BTB integrity. In conclusion, the novel role played by Sertoli cells in the male reproductive tract may facilitate asymptomatic persistence of EBOV. We also recognize limitations in our investigation that should be considered. Leydig and Sertoli cells were used in this study because the first objective was to establish EBOV tropism for testicular cells as potential reservoirs for viral persistence, but macrophages should be included in future work. Macrophages share phagocytic similarities with Sertoli cells and participate in immune response, but their biological roles differ. Macrophages are motile, phagocytose pathogens, and seek to promote an inflammatory immune response^69^. In comparison Sertoli cells seek to maintain barrier integrity, limit inflammation, phagocytose apoptotic germ cells, and produce metabolic factors for use in spermatogenesis^51^. Future studies comparing the two phagocytes would facilitate identification of novel ways in which Sertoli cells respond to EBOV infection. The longitudinal nature of this study was intended to establish viral replication kinetics and persistence in testicular cells, but a time point for sample collection earlier than 1 dpi would have provided additional support for the results of our EBOV trVLP experiments. In addition, the delayed replication of EBOV in Sertoli cells meant that their survival may have been due in part to our experiment ending too soon for the effects of extended high viral load on Sertoli cells to be observed. This concern is mitigated by our findings that Sertoli cells appear to respond to high viral loads by increasing expression of survival factors, but extended time points may be advisable.Finally, Sertoli cell function is dependent on signaling from cells in the interstitial space such as Leydig cells, and from developing germ cells in the BTB and seminiferous tubule lumen^70^. There are 14 stages of spermatogenesis, during which Sertoli cells routinely shift metabolic activity, morphological configuration, and extracellular signaling activity^51^. We did not consider it feasible to account for the effects each stage of spermatogenesis might have on EBOV replication kinetics and host cell response.

## Supplementary information

Supplementary table 1

## Data Availability

The transcriptomic data analysed during the current study are available in the NCBI Short Read Archive as part of BioProject PRJNA1251592.

## References

[1] Thorson, A. et al. Persistence of Ebola virus in semen among Ebola virus disease survivors in Sierra Leone: A cohort study of frequency, duration, and risk factors. *PLoS Med.* **18**, e1003273 (2021). 33566817 10.1371/journal.pmed.1003273 PMC7875361

[2] Thorson, A., Formenty, P., Lofthouse, C. & Broutet, N. Systematic review of the literature on viral persistence and sexual transmission from recovered Ebola survivors: evidence and recommendations. *BMJ Open* **6**, e008859 (2016). 26743699 10.1136/bmjopen-2015-008859 PMC4716240

[3] Schindell, B. G., Webb, A. L. & Kindrachuk, J. Persistence and sexual transmission of filoviruses. *Viruses* **10**, 683 (2018). 30513823 10.3390/v10120683 PMC6316729

[4] Pandey, A. et al. Strategies for containing Ebola in west Africa. *Science* **346**, 991–995 (2014). 25414312 10.1126/science.1260612 PMC4316831

[5] Bausch, D. G. et al. Assessment of the risk of Ebola virus transmission from bodily fluids and fomites. *J. Infect. Dis.* **196**, S142–S147 (2007). 17940942 10.1086/520545

[6] Dowell, S. F. et al. Transmission of Ebola hemorrhagic fever: a study of risk factors in family members, Kikwit, Democratic Republic of the Congo, 1995. *J. Infect. Dis.* **179**, S87–S91 (1999). 9988169 10.1086/514284

[7] Sissoko, D. et al. Persistence and clearance of Ebola virus RNA from seminal fluid of Ebola virus disease survivors: a longitudinal analysis and modelling study. *Lancet Global Health* **5**, e80–e88 (2017). 27955791 10.1016/S2214-109X(16)30243-1

[8] Barnes, K. G. et al. Evidence of Ebola virus replication and high concentration in semen of a patient during recovery. *Clin. Infect. Dis.* **65**, 1400–1403 (2017). 28582513 10.1093/cid/cix518 PMC5850519

[9] Diallo, B. et al. Resurgence of Ebola virus disease in Guinea linked to a survivor with virus persistence in seminal fluid for more than 500 days. *Clin. Infect. Dis.* **63**, 1353–1356 (2016). 27585800 10.1093/cid/ciw601 PMC5091350

[10] Mate, S. E. et al. Molecular evidence of sexual transmission of Ebola virus. *N. Engl. J. Med.* **373**, 2448–2454 (2015). 26465384 10.1056/NEJMoa1509773 PMC4711355

[11] Arias, A. et al. Rapid outbreak sequencing of Ebola virus in Sierra Leone identifies transmission chains linked to sporadic cases. Virus. *Evolution* **2**, vew016 (2016). 10.1093/ve/vew016 PMC5499387 28694998

[12] Wilson, H. W. et al. Post-Ebola syndrome among Ebola virus disease survivors in Montserrado County, Liberia 2016. *BioMed Res. Int.* **2018**, 1909410 (2018). 30050920 10.1155/2018/1909410 PMC6046154

[13] Deen, G. F. et al. Ebola RNA persistence in semen of Ebola virus disease survivors. *N. Engl. J. Med.* **377**, 1428–1437 (2017). 26465681 10.1056/NEJMoa1511410 PMC5798881

[14] Perry, D. L. et al. Ebola virus localization in the macaque reproductive tract during acute Ebola virus disease. *Ame. J. Pathol.* **188**, 550–558 (2018). 10.1016/j.ajpath.2017.11.004 PMC5840485 29429544

[15] Liu, D. X. et al. Expanded histopathology and tropism of Ebola virus in the rhesus macaque model: Potential for sexual transmission, altered adrenomedullary hormone production, and early viral replication in liver. *Am. J. Pathol.* **192**, 121–129 (2022). 34626576 10.1016/j.ajpath.2021.09.009 PMC8759036

[16] Davis, K. J. et al. Pathology of experimental Ebola virus infection in African green monkeys. *Arch. Pathol. Lab. Med.* **121**, 805–819 (1997). 9278608

[17] Zeng, X. et al. Identification and pathological characterization of persistent asymptomatic Ebola virus infection in rhesus monkeys. *Nat. Microbiol.* **2**, 1–11 (2017). 10.1038/nmicrobiol.2017.113 28715405

[18] Clancy C. S., et al. Establishing a Mouse Model for Sexual Transmission and Male Reproductive Tract Persistence of Ebola virus. *J. Infect. Dis.* **228**, S554–S558 (2023). 10.1093/infdis/jiad118 PMC10651199 37102262

[19] Hoenen, T., Watt, A., Mora, A. & Feldmann, H. Modeling the lifecycle of Ebola virus under biosafety level 2 conditions with virus-like particles containing tetracistronic minigenomes. *J. Vis. Exp. JoVE* **27**, 52381 (2014). 10.3791/52381 PMC4828136 25285674

[20] Zhou, R. et al. The roles and mechanisms of Leydig cells and myoid cells in regulating spermatogenesis. *Cell. Mol. Life Sci.* **76**, 2681–2695 (2019). 30980107 10.1007/s00018-019-03101-9 PMC11105226

[21] Meinhardt, A. & Hedger, M. P. Immunological, paracrine and endocrine aspects of testicular immune privilege. *Mol. Cell. Endocrinol.* **335**, 60–68 (2011). 20363290 10.1016/j.mce.2010.03.022

[22] Stanton, P. G. Regulation of the blood-testis barrier. *Semin. Cell Dev. Biol.* **59**, 166–173 (2016). 10.1016/j.semcdb.2016.06.018 27353840

[23] Nakanishi, Y. & Shiratsuchi, A. Phagocytic removal of apoptotic spermatogenic cells by Sertoli cells: mechanisms and consequences. *Biol. Pharmaceutical Bull.* **27**, 13–16 (2004). 10.1248/bpb.27.13 14709891

[24] Wong, G. et al. Naturally occurring single mutations in Ebola virus observably impact infectivity. *J. Virol.* **93**, 01098–18 (2019). 10.1128/JVI.01098-18 PMC6288345 30333174

[25] Watt, A. et al. A novel life cycle modeling system for Ebola virus shows a genome length-dependent role of VP24 in virus infectivity. *J. Virol.* **88**, 10511–10524 (2014). 24965473 10.1128/JVI.01272-14 PMC4178905

[26] Bolger, A. M., Lohse, M. & Usadel, B. Trimmomatic: a flexible trimmer for Illumina sequence data. *Bioinformatics* **30**, 2114–2120 (2014). 24695404 10.1093/bioinformatics/btu170 PMC4103590

[27] Kim, D., Langmead, B. & Salzberg, S. L. HISAT: a fast spliced aligner with low memory requirements. *Nat. Methods* **12**, 357–360 (2015). 25751142 10.1038/nmeth.3317 PMC4655817

[28] Liao, Y., Smyth, G. K. & Shi, W. featureCounts: an efficient general purpose program for assigning sequence reads to genomic features. *Bioinformatics* **30**, 923–930 (2014). 24227677 10.1093/bioinformatics/btt656

[29] Love, M. I., Huber, W. & Anders, S. Moderated estimation of fold change and dispersion for RNA-seq data with DESeq2. *Genome Biol.* **15**, 1–21 (2014). 10.1186/s13059-014-0550-8 PMC4302049 25516281

[30] Zhou, Y. et al. Metascape provides a biologist-oriented resource for the analysis of systems-level datasets. *Nat. Commun.* **10**, 1523 (2019). 30944313 10.1038/s41467-019-09234-6 PMC6447622

[31] Gillespie, M. et al. The reactome pathway knowledgebase 2022. *Nucleic Acids Res.* **50**, D687–D692 (2022). 34788843 10.1093/nar/gkab1028 PMC8689983

[32] Thomas, P. D. et al. PANTHER: Making genome‐scale phylogenetics accessible to all. *Protein Sci.* **31**, 8–22 (2022). 34717010 10.1002/pro.4218 PMC8740835

[33] Ashburner, M. et al. Gene ontology: tool for the unification of biology. *Nat. Genet.* **25**, 25–29 (2000). 10802651 10.1038/75556 PMC3037419

[34] Aleksander, S. A. et al. The Gene Ontology knowledgebase in 2023. *Genetics* **224**, iyad031 (2023). 36866529 10.1093/genetics/iyad031 PMC10158837

[35] Li, Y. et al. A systematic approach for analysis of peptide array kinome data. *Sci. Signal.* **5**, pl2–pl2 (2012). 22510468 10.1126/scisignal.2002429

[36] Kindrachuk, J. et al. Ebola virus modulates transforming growth factor β signaling and cellular markers of mesenchyme-like transition in hepatocytes. *J. Virol.* **88**, 9877–9892 (2014). 24942569 10.1128/JVI.01410-14 PMC4136307

[37] Huber, W., Von Heydebreck, A., Sültmann, H., Poustka, A. & Vingron, M. Variance stabilization applied to microarray data calibration and to the quantification of differential expression. *Bioinformatics* **18**, S96–S104 (2002). 12169536 10.1093/bioinformatics/18.suppl_1.s96

[38] Trost, B., Kindrachuk, J., Määttänen, P., Napper, S. & Kusalik, A. PIIKA 2: an expanded, web-based platform for analysis of kinome microarray data. *PloS One* **8**, e80837 (2013). 24312246 10.1371/journal.pone.0080837 PMC3843739

[39] Srinivasan, B. et al. TEER measurement techniques for in vitro barrier model systems. *J. Lab. Autom.* **20**, 107–126 (2015). 25586998 10.1177/2211068214561025 PMC4652793

[40] Benson, K., Cramer, S. & Galla, H.-J. Impedance-based cell monitoring: barrier properties and beyond. *Fluids Barriers CNS* **10**, 1–11 (2013). 23305242 10.1186/2045-8118-10-5 PMC3560213

[41] Shi, J. F. et al. Characterization of cholesterol metabolism in Sertoli cells and spermatogenesis (Review). *Mol. Med. Rep.* **17**, 705–713 (2018). 10.3892/mmr.2017.8000 PMC5780145 29115523

[42] Shen, W.-J., Azhar, S. & Kraemer, F. B. SR-B1: a unique multifunctional receptor for cholesterol influx and efflux. *Ann. Rev. Physiol.* **80**, 95–116 (2018). 29125794 10.1146/annurev-physiol-021317-121550 PMC6376870

[43] Nikitina, E., Larionova, I., Choinzonov, E. & Kzhyshkowska, J. Monocytes and macrophages as viral targets and reservoirs. *Int. J. Mol. Sci.* **19**, 2821 (2018). 30231586 10.3390/ijms19092821 PMC6163364

[44] Washburn, R. L., Hibler, T., Kaur, G. & Dufour, J. M. Sertoli cell Immune Regulation: a double-edged Sword. *Front. Immunol.* **13**, 913502 (2022). 35757731 10.3389/fimmu.2022.913502 PMC9218077

[45] Pleet, M. L. et al. Ebola virus VP40 modulates cell cycle and biogenesis of extracellular vesicles. *J. Infect. Dis.* **218**, S365–S387 (2018). 30169850 10.1093/infdis/jiy472 PMC6249571

[46] Bavari, S. et al. High Content Image Based Analysis Identifies Cell Cycle Inhibitors as Regulators of Ebola Virus. *Infection.* **4**, 1865–1877 (2012). 10.3390/v4101865 PMC3497033 23202445

[47] Han, Z., Licata, J. M., Paragas, J. & Harty, R. N. Permeabilization of the plasma membrane by Ebola virus GP2. *Virus Genes* **34**, 273–GP281 (2007). 16927113 10.1007/s11262-006-0009-4

[48] Theocharis, A. D., Skandalis, S. S., Gialeli, C. & Karamanos, N. K. Extracellular matrix structure. *Adv. Drug Deliv. Rev.* **97**, 4–27 (2016). 26562801 10.1016/j.addr.2015.11.001

[49] Misasi, J. & Sullivan, N. J. Camouflage and misdirection: the full-on assault of ebola virus disease. *Cell* **159**, 477–486 (2014). 25417101 10.1016/j.cell.2014.10.006 PMC4243531

[50] Xiao, X., Mruk, D. D., Wong, C. K. & Yan Cheng, C. Germ cell transport across the seminiferous epithelium during spermatogenesis. *Physiology* **29**, 286–298 (2014). 24985332 10.1152/physiol.00001.2014 PMC4103058

[51] Kaur, G., Thompson, L. A. & Dufour, J. M. Sertoli cells–immunological sentinels of spermatogenesis. *Semin. Cell Dev. Biol.* ***30****,* 36–44 (2014). 10.1016/j.semcdb.2014.02.011 PMC4043859 24603046

[52] Kafri, P. et al. Quantifying β-catenin subcellular dynamics and cyclin D1 mRNA transcription during Wnt signaling in single living cells. *Elife* **5**, e16748 (2016). 27879202 10.7554/eLife.16748 PMC5161448

[53] Jankeel, A. et al. Early transcriptional changes within liver, adrenal gland, and lymphoid tissues significantly contribute to ebola virus pathogenesis in cynomolgus macaques. *J. Virol.* **94**, 00250–20 (2020). 10.1128/JVI.00250-20 PMC7269430 32213610

[54] Price, A. et al. Transcriptional correlates of tolerance and lethality in mice predict Ebola virus disease patient outcomes. *Cell Rep.* **30**, 1702–1713.e6 (2020). 32049004 10.1016/j.celrep.2020.01.026 PMC11062563

[55] Liu, X. et al. Transcriptomic signatures differentiate survival from fatal outcomes in humans infected with Ebola virus. *Genome Biol.* **18**, 1–17 (2017). 28100256 10.1186/s13059-016-1137-3 PMC5244546

[56] Kühl, A. & Pöhlmann, S. How Ebola virus counters the interferon system. *Zoonoses Public Health* **59**, 116–131 (2012). 22958256 10.1111/j.1863-2378.2012.01454.x PMC7165950

[57] Kuzmin, I. V. et al. Innate immune responses of bat and human cells to filoviruses: commonalities and distinctions. *J. Virol.* **91**, e02471–16 (2017). 28122983 10.1128/JVI.02471-16 PMC5375674

[58] Tang, H. et al. Ebola virus–like particles reprogram cellular metabolism. *J. Mol. Med.* **101**, 557–568 (2023). 36959259 10.1007/s00109-023-02309-4 PMC10036248

[59] Garnett, L. et al. Adipose Tissues from Human and Bat-Derived Cell Lines Support Ebola Virus Infection. *Viruses* **15**, 1827 (2023). 37766234 10.3390/v15091827 PMC10537186

[60] Oliveira, P. F., Martins, A. D., Moreira, A. C., Cheng, C. Y. & Alves, M. G. The Warburg effect revisited—lesson from the Sertoli cell. *Med. Res. Rev.* **35**, 126–151 (2015). 25043918 10.1002/med.21325 PMC4845724

[61] Kaiser, G. R. et al. Metabolism of amino acids by cultured rat Sertoli cells. *Metabolism* **54**, 515–521 (2005). 15798960 10.1016/j.metabol.2004.11.005

[62] Falasca, L. et al. Molecular mechanisms of Ebola virus pathogenesis: focus on cell death. *Cell Death Differ.* **22**, 1250–1259 (2015). 26024394 10.1038/cdd.2015.67 PMC4495366

[63] Moni, B. M., Sakurai, Y., Yasuda, J. & Ebola Virus, G. P. activates endothelial cells via host cytoskeletal signaling factors. *Viruses* **14**, 142 (2022). 35062347 10.3390/v14010142 PMC8781776

[64] Baseler, L., Chertow, D. S., Johnson, K. M., Feldmann, H. & Morens, D. M. The pathogenesis of Ebola virus disease. *Ann. Rev. Pathol. Mech. Dis.* **12**, 387–418 (2017). 10.1146/annurev-pathol-052016-100506 27959626

[65] Lai, K. Y., Ng, W. Y. G. & Cheng, F. F. Human Ebola virus infection in West Africa: a review of available therapeutic agents that target different steps of the life cycle of Ebola virus. *Infect. Dis. Poverty* **3**, 1–17 (2014). 25699183 10.1186/2049-9957-3-43 PMC4334593

[66] Younan, P., Iampietro, M. & Bukreyev, A. Disabling of lymphocyte immune response by Ebola virus. *PLoS Pathogens* **14**, e1006932 (2018). 29649305 10.1371/journal.ppat.1006932 PMC5897007

[67] Guerriero, G., Trocchia, S., Abdel-Gawad, F. K. & Ciarcia, G. Roles of reactive oxygen species in the spermatogenesis regulation. *Front. Endocrinol.* **5**, 56 (2014). 10.3389/fendo.2014.00056 PMC4001055 24795696

[68] Yang, Y., Bazhin, A. V., Werner, J. & Karakhanova, S. Reactive oxygen species in the immune system. *Int. Rev. Immunol.* **32**, 249–270 (2013). 23617726 10.3109/08830185.2012.755176

[69] McNelis, J. C. & Olefsky, J. M. Macrophages, immunity, and metabolic disease. *Immunity* **41**, 36–48 (2014). 25035952 10.1016/j.immuni.2014.05.010

[70] Ni, F.-D., Hao, S.-L. & Yang, W.-X. Multiple signaling pathways in Sertoli cells: recent findings in spermatogenesis. *Cell Death Dis.* **10**, 541 (2019). 31316051 10.1038/s41419-019-1782-z PMC6637205

[71] Shannon, P. et al. Cytoscape: a software environment for integrated models of biomolecular interaction networks. *Genome Res.* **13**, 2498–2504 (2003). 14597658 10.1101/gr.1239303 PMC403769

